# A Pediatric Patient With Seizures and Vagus Nerve Stimulation With Worsening Snoring and Apneas

**DOI:** 10.7759/cureus.14379

**Published:** 2021-04-09

**Authors:** Sameh S Morkous

**Affiliations:** 1 Pediatric Neurology, Lehigh Valley Health Network (LVHN) Lehigh Valley Reilly Children's Hospital, Allentown, USA

**Keywords:** seizures, obstructive sleep apnea, vagus nerve stimulation

## Abstract

An 11-year-old female presents to the sleep clinic for evaluation for possible sleep-disordered breathing (SDB). She has a history of frequently intractable seizures for which she was tried on multiple antiepileptic medications. She had vagus nerve stimulation (VNS) implantation two years ago to treat her focal seizures. Nine months later, her seizures were controlled, but the family raised concerns about louder snoring and more frequently witnessed apneas. She had polysomnography (PSG) that showed severe obstructive sleep apnea (OSA) related to her VNS. The patient was diagnosed with SDB secondary to electrical activations of the implanted VNS. We describe an epilepsy patient whose case illustrates the possible respiratory complications (primarily OSA) associated with VNS. We will discuss the possible mechanisms of VNS related SDB and the importance of screening for SDB and advocate for a PSG both before and after VNS implantation.

## Introduction

Vagus nerve stimulation (VNS) can worsen sleep-disordered breathing (SDB) after implantation. Patients with VNS can have central apneas, obstructive hypopneas, and/or obstructive apneas. Patients with VNS often have an increase in apneic events after implantation [1]. This case highlights the importance of evaluating for this possible VNS side effect and discussing warning signs with the family prior to the VNS implantation. This case will also demonstrate the continuous need for screening prior to and post-VNS implantation.

## Case presentation

An 11-year-old female presented to the sleep clinic for evaluation of snoring and witnessed apneas. She has had a history of focal epilepsy since age four years and was tried on multiple antiseizure medications, including levetiracetam, clobazam, zonisamide, and valproate. Her most recent antiepileptic medications include clonazepam, lacosamide, and topiramate for the last three years. She continued to have up to 10 seizures every week, and so two years ago, the VNS was implanted to manage her intractable epilepsy. Nine months following her VNS implantation her seizures were reduced dramatically, but her mother raised concerns about more frequent nocturnal respiratory pauses during her sleep as well as louder snoring. This was reported by the family for the first time-only after her VNS was implanted. Thus, the patient was referred to our Pediatric Sleep Medicine clinic for an initial evaluation. Epworth Sleepiness Scale (ESS) assessment was six of 24. She had a consistently regular sleep/wake cycle with 10 hours of sleep with a bedtime of 9:30 PM. She usually would fall asleep within 15 minutes and would wake up between 7:30 AM and 8:00 AM. She had no daily naps. The patient had no other significant medical history. Family history included paternal sleep apnea and maternal generalized seizures.

Examination revealed patient weight at 34.8 kg; height, 127.5 cm; Z-scores of -0.89 and -3.05 were based on the Centers for Disease Control (CDC) two to 20 years’ weight-for-age and stature-for-age data, respectively. Body mass index was 21.43 kg/m^2^ (Z-score: 0.98, 84th percentile). She had a Mallampati score of 1; tonsil size of 0. The remainder of the physical examination was normal.

**Figure 1:**
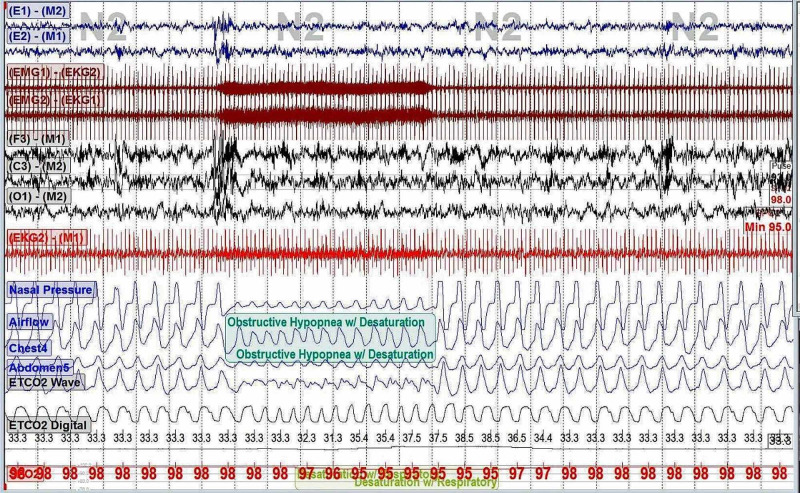
PSG that shows an obstructive hypopnea. PSG: polysomnography

**Figure 2:**
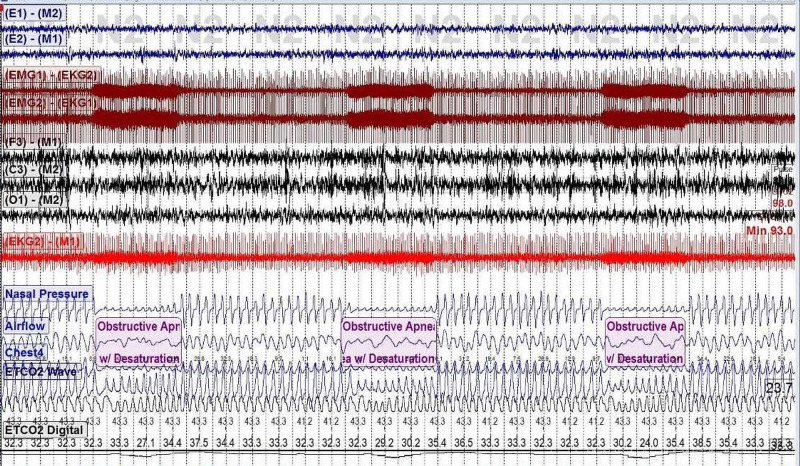
PSG that shows the obstructive apneas. PSG: polysomnography

A nocturnal PSG revealed total sleep time was 467.3 minutes; lights-off time, 10 PM; lights-on time, 6 AM. The obstructive apnea-hypopnea index (O-AHI) was 30.7 events/hour; central apnea index, 0.1 events/hour. Sleep latency was five minutes, lowest oxygen saturation was 90%, and mean end-tidal CO_2_ (EtCO_2_) was 33 mmHg. Periodic limb movement index was 1.0 events/hour. The patient’s polysomnography (PSG) demonstrates the respiratory events (Figures 1, 2).

Differential diagnosis

The patient has evidence of severe obstructive sleep apnea (OSA) on the PSG. Respiratory events were unusually rhythmic and occurred with the accuracy of a metronome [2]. Each respiratory event lasted exactly 30 seconds and was separated from the next event by five minutes. Upon careful examination of the polysomnographic signals, an artifact of high frequency was seen on the electromyogram (EMG) and the electrocardiogram (EKG) leads. The artifact occurred in bursts, each lasting 30 seconds. The occurrence of this artifact occurred in sync with the patient’s respiratory events.

These findings on the polysomnographic signals would raise questions about their possible etiologies that can include possibilities like an underlying arrhythmia or an artifact not related to the VNS versus a VNS related phenomena. Recognizing the exact etiology and accurately answering these questions is pivotal to decide what will be the best next step for management.

Questions and answers

The findings on the polysomnographic signals would raise questions about their possible etiologies that can include possibilities like an underlying arrhythmia or an artifact not related to the VNS versus a VNS related phenomena. Recognizing the exact etiology and accurate interpretation of these findings is pivotal to decide what will be the best next step for management. 

1. What is the most possible etiology of the electromyogram (EMG) and the electrocardiogram (EKG) artifacts in the figures above?

(A) Myocardial fiber stretch during episodes of apnea; (B) electrical activations of the implanted VNS; (C) undiagnosed supraventricular arrhythmia; (D) ungrounded recording equipment affected by a nearby air-conditioner [2].

The answer is B. Electrical activations of the implanted VNS. (The figures above outlines the EMG and the EKG electrical artifacts coinciding with the patient’s respiratory event in 30-second bursts. This regular and repetitive pattern argues against options {A} and {D}. There is no concern for arrhythmia on the ECG lead which argues against option {C}.)

2. What will be the most appropriate next step to address this finding?

These respiratory events can be reduced with changes in the VNS operational parameters or with the use of continuous positive airway pressure (CPAP). (This will be discussed further below in detail.)

Treatment

The patient had a repeat PSG for positive airway pressure (PAP) therapy. Her repeat PSG one month later with the VNS “on” showed an optimal pressure of 7 cm H_2_0 during which the (O-AHI) was 1.3. The patient was started on PAP therapy. The VNS could have also worsened a preexisting OSA and thus the patient was also referred to ear, nose, and throat (ENT) for evaluation of possible surgical options like Tonsillectomy and Adenoidectomy (T&A). The ENT evaluation did not identify any additional surgical options.

Outcome and follow-up

On the three-month follow-up after PAP therapy was started the patient tolerated the PAP therapy well with 99.3% of days usage-an average usage of seven hours and 51 minutes per night and 90% of days with usage >=4 hours. There were no reported side effects to PAP therapy. The patient remained seizure-free for the last year and is currently on clonazepam and lacosamide.

## Discussion

There is a paucity of cases demonstrating this finding in children. A robust PubMed database search for the search terms VNS & sleep apnea yielded 69 articles on this topic, most of these articles were discussing either the VNS role in epilepsy and/ or the respiratory complications associated with vagus nerve stimulators, however, only one article matched citation for VNS and obstructive sleep apnea in adults. Also, a systematic review on the topic has come to light by Óscar Romero-Osorio et al. that describes only four case reports in adults between the ages of 20 years and 57 years at the time of the evaluation in the United States [3]. A similar PubMed search in pediatrics yielded 11 articles with only one systematic review of the literature describing the comorbidity of sleep apneas and epilepsy in childhood [4] but with only one case report [5]. The effects of vagal nerve stimulation on sleep-related breathing have not been well-described in children and there is an overall lack of case reports, especially in children, demonstrating respiratory complications (primarily OSA) associated with VNS [6].

Few case reports in the literature have reported a possible association between the VNS and the respiratory events. Shaman reported a 50-year-old male who presented with snoring, witnessed apneas, unrefreshing sleep, and excessive daytime sleepiness (ESS 17) despite adequate sleep duration [2]. This patient had a history of hypertension (well controlled with amlodipine and hydrochlorothiazide) and a seizure disorder that was difficult to treat. The patient continued to have seizures (about twice a week) while taking phenytoin, lamotrigine, and levetiracetam. A VNS was implanted one year prior to the presentation, which resulted in the reduction of seizure frequency to twice per month. A PSG confirmed the presence of mild OSA (apnea/hypopnea index 13.5). However, the respiratory events were unusually rhythmic and occurred with the accuracy of a metronome. This case showed electrical activations related to the implanted VNS but this patient was an adult and the use of PAP therapy was not discussed.

 Recently, an abstract by Ochoa et al. described a 12-year-old female with medically intractable epilepsy who had a VNS implantation as an additional therapy [7]. She was referred for snoring, excessive tiredness, and frequent movements during sleep. The Pediatric Daytime Sleepiness Scale (PDSS) was 11. During the overnight PSG, there was evidence of obstructive apneas and hypopneas associated with the VNS firing (30-seconds on and 180-seconds off). The patient had an O-AHI of 13.5 events/hour, oxygen nadir of 77%, and partial pressure of carbon dioxide (PCO_2_) of 41. Bilevel positive airway pressure (BiPAP) was initiated and titrated to 11/7 cm H_2_0 with a backup rate of 12. BiPAP with the VNS “on” resulted in an O-AHI of 0.0 and oxygen nadir of 94%. This case, however, did not diagrammatically illustrate the VNS-related respiratory events similar to our case and did not emphasize the importance of screening before and after the VNS implantation. The AHI was also much higher in our case (13.5 events/hour versus 30.7 events/hour in this case presented). Our case adds to the existing literature and raises awareness about the importance of screening for OSA and SDB even before the VNS is implanted.

Intermittent VNS can reduce the frequency of seizures in patients with refractory epilepsy. Although the exact mechanism of seizure reduction remains unclear, Mithani, et al. reported that the vagus afferent network (VagAN) is thought to be the neural substrate of VNS efficacy. This recent study provides the first multi-institutional, multimodal, connectomic prediction algorithm to reliably identify VNS responders based on the reported group differences in their white matter microstructure that was demonstrated on the diffusion tensor imaging used in this study. This is important in order to mitigate surgical risks for those children predicted not to benefit from the VNS implantation and to ensure cost-effective allocation of health care resources [8]. Stimulation of vagus nerve afferent fibers can also cause vocal cord dysfunction, laryngeal spasm, cough, dyspnea, nausea, and vomiting. Another reported side effect of VNS activations is SDB. The VNS activations cause spasms in the upper airway muscles resulting in obstructive apneas. VNS causes an increase in respiratory rate and decreases in respiratory amplitude, tidal volume, and oxygen saturation during periods of device activation. It usually does not cause arousal or a change in heart rate or blood pressure. Most patients have an increase in their apnea-hypopnea index (AHI). These respiratory events can be reduced with changes in the VNS operational parameters or with the use of CPAP [1].

Manipulating VNS settings such as stimulus intensity, frequency, and cycle times may decrease the respiratory disturbance [5,9]. However, the specific stimulus parameter adjustment that would be most effective in ameliorating the VNS-related respiratory arrhythmia is not known at this time [9]. Our patient had reported snoring and witnessed apneas prior to the VNS implantation that worsened after the VNS was implanted, and she was found to have severe OSA on the PSG. Thus, she could have had a baseline mild or moderate OSA prior to the VNS implantation that worsened after the VNS was implanted. If a pre-VNS implantation PSG has been done, her OSA could have been identified and addressed prior to the VNS surgery. The VNS can have beneficial effects on sleep. This was reported where the VNS was found to induce a significant increase in slow-wave sleep (SWS) and a decrease in sleep latency and stage one sleep in 15 children with intractable epilepsy following VNS stimulation in these children. Sleep parameters including all-night delta power activity and movement times (MTs), used to account for arousals, were estimated in this study where the number and the density of MTs during total night sleep were significantly increased in this analysis. This study reported a significant increase in the number of MTs immediately related to the VNS stimulation periods with a reduction in the epileptiform activity, the clinical seizures, and with improvement in quality of life (QOL) and behavior [10]. The VNS may also interfere with effective CPAP titration. A case study to examine the effects of VNS cycling on CPAP titration for OSA in a 54-year-old male patient with medically intrac­table epilepsy found that adequate CPAP titration could not be achieved in the presence of the patient’s standard VNS on/off cycling mode. However, when this patient was restudied with his VNS device turned off, a nasal CPAP pressure of 13 cm H_2_O resulted in the effective treatment of his severe OSA. This study also suggests PSG before VNS implantation should be considered in order to identify pa­tients with OSA [11]. A similar finding was also reported by Oh et al. who described a case series of four patients with VNS who underwent PSG concurrently with VNS stimulation monitoring and adjustment as well as PAP treatment. This study provided additional evidence of VNS-induced SDB as a side effect of the VNS and highlighted that screening of primary OSA before and after VNS implant is crucial. In addition, PAP treatment alone was not effective in eliminating the VNS-induced SDB in this study and the VNS setting titration showed a dose-dependent effect on SDB [12].

There are complex relationships between epilepsy and OSA. Patients with refractory epilepsy need assessment for undiagnosed and untreated SDB before implantation of VNS devices. Patients with VNS often have an increase in their apneic events after implantation, and these patients need screening for sleep apnea before and after implantation [1].

## Conclusions

This case demonstrates the importance of screening for SDB, preferably before, but also after the VNS implantation. This case illustrates these concepts, especially in children, where scarce cases are describing these findings. We also elaborate on the possible mechanisms of VNS related SDB and discuss the variable management options that the providers can choose to pursue like CPAP utilization or possible changes in the VNS operational application to address the medical condition.

Additional research including a larger group of patients is needed in order to conduct a subgroup analysis to determine the concrete clinical practice guidelines of which subgroup of patients for which routine screening for SDB and recommending the PSG either before or after the VNS implantation or both will be most beneficial. This case raises awareness about this phenomenon and serves as a valuable proponent in the literature for providers to consider screening for SDB as well as a PSG both before and after the VNS implantation.
